# Development of multiplex PCR for neglected infectious diseases

**DOI:** 10.1371/journal.pntd.0007440

**Published:** 2019-07-08

**Authors:** Nutchanart Sea-liang, Amornpun Sereemaspun, Kanitha Patarakul, Jariyanart Gaywee, Wuttikon Rodkvamtook, Nattachai Srisawat, Supaporn Wacharaplusadee, Thiravat Hemachudha

**Affiliations:** 1 Nanomedicine Research Unit, Department of Anatomy, Faculty of Medicine, Chulalongkorn University, Bangkok, Thailand; 2 Department of Microbiology, Faculty of Medicine, Chulalongkorn University, Bangkok, Thailand; 3 Armed Forces Research Institute of Medical Science, Royal Thai Army, Bangkok, Thailand; 4 Nephrology Unit, Department of Medicine, Faculty of Medicine, Chulalongkorn University, Bangkok, Thailand; 5 Thai Red Cross Emerging Infectious Diseases-Health Science Centre, World Health Organization Collaborating Centre for Research and Training on Viral Zoonoses, King Chulalongkorn Memorial Hospital, Faculty of Medicine, Chulalongkorn University, Bangkok, Thailand; Lowell General Hospital, UNITED STATES

## Abstract

Scrub typhus, murine typhus, and leptospirosis are widely neglected infectious diseases caused by *Orientia tsutsugamushi*, *Rickettsia typhi*, and pathogenic *Leptospira* spp., respectively. Patients usually present with non-specific symptoms and therefore are commonly diagnosed with acute undifferentiated febrile illness. Consequently, patients face delayed treatment and increased mortality. Antibody-based serological test currently used as gold standard has limitations due to insufficient antibody titers, especially in the early phase of infection. In this study, we aimed to develop multiplex PCR to combine 3 primer pairs that target specific genes encoding 56-kDa TSA of *O*. *tsutsugamushi*, 17-kDa antigen of *R*. *typhi*, and LipL32 of *L*. *Interrogans* and evaluate its performance in comparison to the standard serological tests. Using EDTA blood samples of known patients, the sensitivity and specificity of our multiplex PCR was 100% and 70%, respectively. In addition, the assay was able to diagnose the co-infection of scrub typhus and leptospirosis. The assay may be useful in identifying causative agents during the early phase of these diseases, enabling prompt and appropriate treatment.

## Author summary

Scrub typhus, murine typhus, and leptospirosis are diagnosed as acute undifferentiated febrile illness. Diagnostic tests for these diseases depend on antibody detection. However, antibody detection is still limited by its tendency to return negative results during the early phase of aforementioned diseases. In this study, a novel multiplex PCR has been developed for detecting *Orientia tsutsugamushi*, *Rickettsia typhi*, and *Leptospira interrogans* that are simultaneously amplified in a single tube. The results have shown that multiplex PCR could be used as a diagnostic tool for detecting bacteria during the early phase of scrub typhus, murine typhus, and leptospirosis, allowing for administration of appropriate treatment.

## Introduction

Scrub typhus, murine typhus, and leptospirosis are widely neglected infectious diseases, especially in the tropical and temperate climate regions, caused by *Orientia tsutsugamushi*, *Rickettsia typhi*, and pathogenic *Leptospira* spp., respectively. *Orientia* and *Rickettsia* are obligate intracellular bacteria. *O*. *tsutsugamushi* is transmitted to human through chigger bite, and *R*. *typhi* infection is transmitted by inoculation of rat flea’s feces on human skin. Pathogenic leptospires are spirochetal bacteria that are mainly transmitted through abraded skin or mucosa after contact with urine of infected animals, contaminated water, or contaminated soil [1–5]. Unfortunately, current diagnostic tools, together with awareness and experience of physicians, have been the limited.

Early clinical manifestations of scrub typhus, murine typhus, and leptospirosis, such as high fever, headache, muscular pain, and anorexia, are non-specific and usually diagnosed as acute undifferentiated febrile illness. These clinical manifestations range from mild, severe, to possibly fatal [6–8]. Eschar caused by chigger bite is a clinical appearance of scrub typhus; however, it is not always present. Moreover, eschar-like lesion can occur in other diseases such as rickettsialpox and anthrax [7, 9, 10]. Macular rash is generally present in murine typhus and scrub typhus as well [11]. The clinical manifestations of leptospirosis are biphasic fever and multi-organ failure in severe cases (Weil’s disease) [12]. Due to clinical manifestations of these diseases being recognized as acute undifferentiated febrile illness and subsequently underdiagnosed, a rapid and reliable laboratory investigation is necessary for confirmation and treatment efficiency.

Standard diagnostic tests for scrub typhus and murine typhus, i.e. indirect immunofluorescence assay (IFA), and for leptospirosis, i.e. microscopic agglutination test (MAT), mainly depend on detection of antibodies which often return false negative results in the early phase of diseases. To identify pathogen in the early phase of disease, direct pathogen identification is of vital importance since these bacteria can be found in the bloodstream during the first week after onset [13, 14]. Thus, a new diagnostic test development based on antigen or DNA detection is essential for rapid diagnosis and proper treatment.

Multiplex PCR is a molecular laboratory test used for simultaneous amplification that utilizes different primers in a single tube [15]. It has been applied in diagnosis of several infectious diseases caused by bacteria, fungi, parasites, and viruses [16]. The multiplex PCR is fast and time-saving because it is capable of detecting multiple pathogens at the same time [17]. In this study, we developed multiplex PCR to detect *O*. *tsutsugamushi*, *L*. *interrogans*, and *R*. *typhi* and evaluated its efficiency compared to serological tests. This work selected three target genes encoding 56-kDa type-specific antigen (TSA) of *O*. *tsutsugamushi*, lipL32 of *L*. *interrogans*, and 17-kDa antigen of *R*. *typhi* to identify each bacterium in bacterial cell culture and blood samples of suspected patients.

## Materials and methods

### Microorganisms

*O*. *tsutsugamushi* (Karp, Kato, and Gilliam strains) and *R*. *typhi* were obtained from Armed Forces Research Institute, Thailand. Pathogenic *L*. *interrogans* (serovar Pyrogenes, Pomona, and Bratislava) and non-pathogenic *L*. *biflexa* serovar Patoc were acquired from Department of Microbiology, Faculty of Medicine, Chulalongkorn University, Thailand.

Other pathogens, consisting of *Escherichia coli*, *Enterococcus faecalis*, *Staphylococcus aureus*, *Klebsiella pneumoniae*, *Salmonella* spp., Dengue virus (serotype1-4), *Plasmodium falciparum*, and *Plasmodium vivax*, were acquired from Department of Microbiology and Department of Parasitology, Faculty of Medicine, Chulalongkorn University, Thailand.

### Clinical samples

This study used 83 EDTA blood samples taken from patients presented with acute undifferentiated febrile illness at Armed Forces Research Institute, King Chulalongkorn Memorial hospital, Loei hospital, Takuapa hospital and Chokchai hospital, Thailand. Patients included were over 18 years old, having acute fever (38°C or higher), exhibiting non-specific symptoms, (headache, muscular pain, anorexia, and rash for 3–5 days), and tested negative for both influenza and dengue antigens. Samples were tested in double-blind experiments and confirmed by IFA, for scrub typhus and murine typhus, and MAT, for leptospirosis. IFA and MAT are antibody detection techniques used as indicator of acute or current exposure; a fourfold increase of antibody titer in paired serum is determined as a positive result [2, 13, 14, 18].

### Ethic statement

This study was approved by Institutional Review Board, Faculty of Medicine, Chulalongkorn University, Thailand (IRB No. 009/57, 534/57, and 380/59). As we had used human EDTA blood obtained from hospital laboratories that perform serology and molecular analysis, an informed consent document was not required. We have not acquired any patient identification, and the data were analyzed anonymously.

### Genomic DNA extraction

Bacterial DNA and blood samples were extracted using PureLink Genomic DNA Mini Kit (Invitrogen, USA). First, bacterial cells were lysed by PureLink Genomic Lysis/Binding Buffer. Then, DNA extraction was carried out according to PureLink manufacturer’s instruction. DNA was eluted in 50μl PureLink Genomic Elution Buffer. DNA concentration was measured by UV absorbance at 260/280.

To isolate DNA from blood samples, each blood sample (200μl per sample) was added to 180μl PureLink Genomic Lysis/Binding Buffer. DNA isolation was carried out according to PureLink manufacturer’s instruction. Isolated DNA was kept at -20°C until use.

### Primer design

The target genes consisted of 56-kDa TSA gene, 17-kDa antigen gene, and *lipL32* gene were selected for this study. 56-kDa TSA (56K TSA_F and 56K TSA_R) primers were designed from accession no. M33004, using Primer 3 Software. 17-kDa (17K_F and 17K_R) primers followed the research conducted by Webb et al. [19]. LipL32 (LipL32_45F and LipL32_287R) primers were slightly modified from that of Bourhy et al. [20]. Primer sequences and their amplicon sizes are shown in Table 1. The specificity of primers was bioinformatically aligned with databases in National Center for Biotechnology Information (NCBI) using BioEdit software.

**Table 1:** List of primer sequences.

Primer name	Sequence (5’→3’)	Length (bp)	Product size (bp)	references
56-kDa_F	GGC-CAA-GTT-AAA-CTC-TAT-GCT-GAC	24	166	-
56-kDa_R	CAG-CAT-TAA-TTG-CTA-CAC-CAA-GTG-C	25		
lipL32_45F	AAG-CAT-TAC-CGC-TTG-TGG-TG	20	243	[20]
lipL32_287R	CGA-ACT-CCC-ATT-TCA-GCG-AT	20		
17-kDa_F	GCT-CTT-GCA-GCT-TCT-ATG-TT	20	434	[19]
17-kDa_R	CAT-TGT-TCG-TCA-GGT-TGG-CG	20		

### Optimization of singleplex and multiplex PCR

Singleplex PCR condition was initially optimized according to GoTaq Flexi manual instruction (Promega, USA). Multiplex PCR was optimized to ensure appropriate amplification. Extension temperatures of 68°C and 72°C were tested in this experiment. Magnesium chloride concentration, ranging from 1.5–4.0 mM, was adjusted to give the most intensity for all PCR products. Annealing temperature, ranging from 55°C-61°C, was optimized using a gradient thermal cycler. PCR reaction was carried out in a total volume of 20μl per reaction. Multiplex PCR amplification was performed on Thermalcycler (Applied Biosystems, USA) under the following conditions; 94°C for 5 min, followed by 35 cycles of 94°C for 30 sec, 55–61°C for 1 min, 68/72°C for 1 min, and final extension at 68°C for 10 min.

### Sensitivity and specificity of multiplex PCR

Limit of detection of multiplex PCR was determined using a tenfold serial dilution, ranging from 5x10^0^ to 5x10^-6^ ng/μl per reaction, of *Orientia*, *Rickettsia* and leptospiral DNA mixture.

Specificity of multiplex PCR was tested with unrelated pathogens such as *Escherichia coli*, *Enterococcus faecalis*, *Staphylococcus aureus*, *Klebsiella pneumonia*, *Salmonella* spp., Dengue virus (serotype1-4), *Plasmodium falciparum*, and *Plasmodium vivax*. The efficiency of this assay was evaluated using masking conditions that mixes *Orientia*, *Rickettsia* or *Leptospira* with other bacteria.

### Performance of multiplex PCR

Multiplex PCR was validated in blood samples of acute undifferentiated febrile illness patients compared to serological methods (either IFA or MAT). Diagnostic accuracy of this assay was measured in term of sensitivity, specificity, positive predictive value, and negative predictive value compared to the gold standard methods. The equations for calculating sensitivity, specificity, positive predictive value, and negative predictive value are shown in Table 2.

**Table 2:** List of the equations for calculating the performance of multiplex PCR.

	Equation
Sensitivity	$\frac{\mathrm{true}\;\mathrm{positive}}{\mathrm{true}\;\mathrm{positive}+\mathrm{false}\;\mathrm{negative}}$
Specificity	$\frac{\mathrm{true}\;\mathrm{negative}}{\mathrm{true}\;\mathrm{negative}+\mathrm{false}\;\mathrm{positive}}$
Positive predictive value	$\frac{\mathrm{true}\;\mathrm{positive}}{\mathrm{true}\;\mathrm{positive}+\mathrm{false}\;\mathrm{positive}}$
Negative predictive value	$\frac{\mathrm{true}\;\mathrm{negative}}{\mathrm{true}\;\mathrm{negative}+\mathrm{false}\;\mathrm{negative}}$

### Gel electrophoresis

After PCR amplification, PCR products were loaded onto 2% agarose gel (Invitrogen, USA) in 0.5X TBE (Tris-borate with EDTA buffer) and separated by horizontal gel electrophoresis for 30 min at 100 volts. Then, agarose gel was stained in 0.5 μg/μl ethidium bromide (Sigma, USA) for 5 min and de-stained in deionized water for 10 min. Amplicons were visualized by UV transilluminator (Biorad, USA).

## Results

### Optimization of singleplex and multiplex PCR

**Fig 1:**
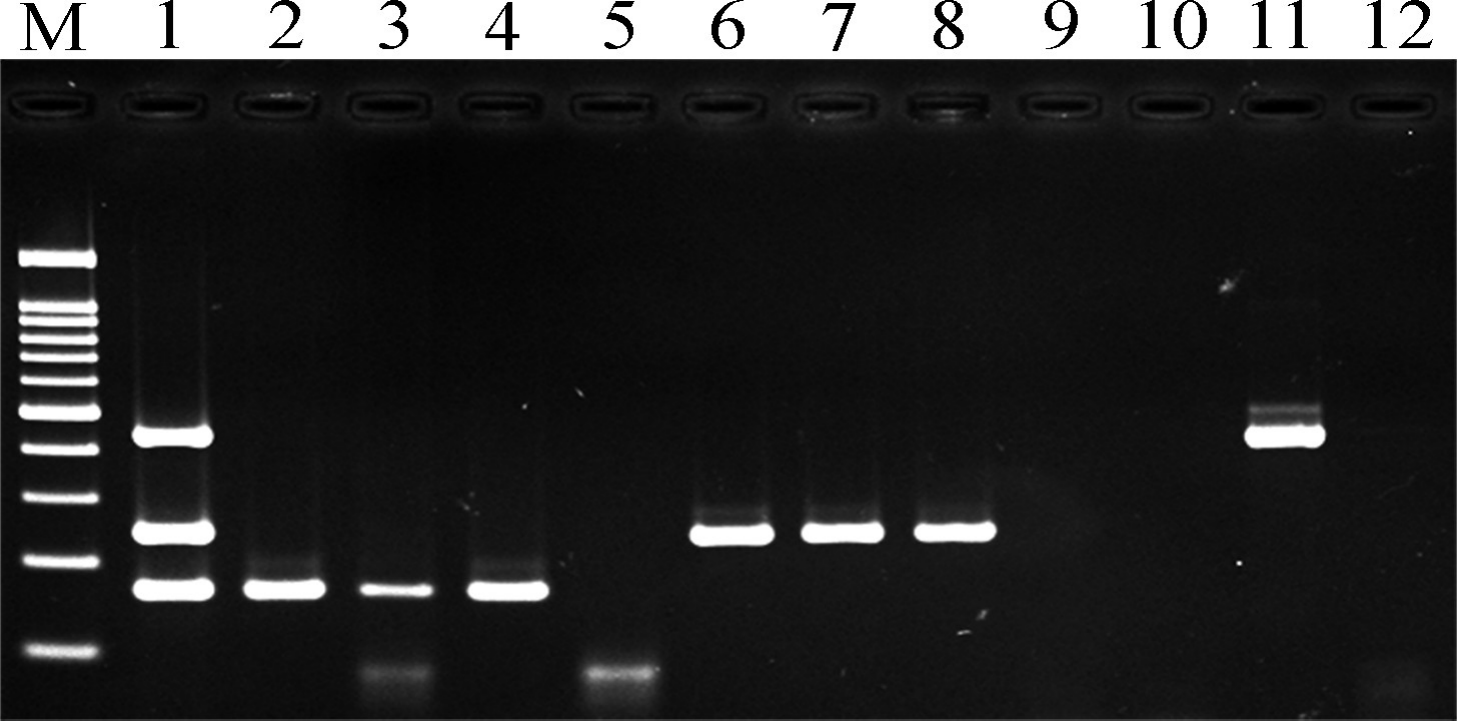
Optimization of singleplex and multiplex PCR. Lane M; 100bp marker, lane 1; DNA mixture derived from *Orientia* (166 bp), *Leptospira* (243 bp), and *Rickettsia* (434 bp) was amplified using multiplex PCR. Lane 2–4; *O*. *tsutsugamushi* (Karp, Kato, and Gilliam strains), lane 5; negative control, lane 6–8; *L*. *interrogans* (serovar Bratislava, Pyrogenes, and Pomona), lane 9; *L*. *biflexa* serovar Patoc, lane 10; negative control, lane 11; *R*. *typhi*, and lane 12; negative control. Lane 2–12; DNA was amplified using singleplex PCR.

In this experiment, optimal conditions were initially determined for singleplex PCR and, subsequently, for the multiplex PCR reaction. The results showed different sizes of PCR products containing a 166-bp fragment of *Orientia* DNA, 434-bp fragment of *Rickettsia* DNA, and 243-bp fragment of leptospiral DNA. In singleplex PCR, the 56-kDa TSA primer set was able to detect all strains of *O*. *tsutsugamushi* (Karp, Kato, and Gilliam strains). 17-kDa antigen primers could amplify DNA of *R*. *typhi*. LipL32 primers amplified pathogenic leptospiral DNA (serovar Bratislava, Pomona, and Pyrogenes), whereas this primer did not amplify non-pathogenic leptospiral DNA (*L*. *biflexa* serovar Patoc). (Fig 1).

Multiplex PCR was adjusted to ensure appropriate amplification of each target. The optimal extension temperature at 68°C gave higher intensity of amplicons than at 72°C. After magnesium chloride concentrations ranging from 1.5–4.0 mM were tested, the optimal concentration of magnesium chloride was found to be 2.5mM. For annealing temperature optimized using gradient PCR, the optimal annealing temperature of 61°C was determined (Fig 1).

### Specificity of multiplex PCR assay

**Fig 2:**
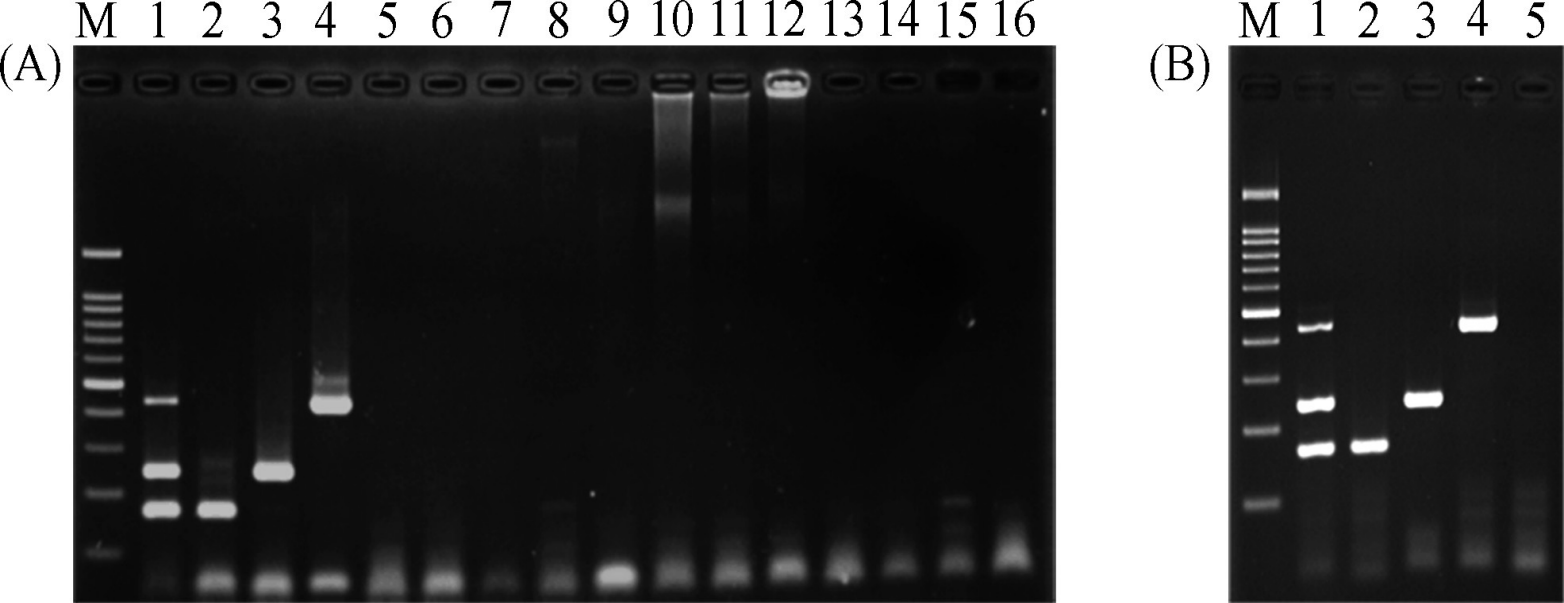
Specificity of multiplex PCR assay. (A) Multiplex PCR was tested with other pathogenic microorganisms. Lane M; 100bp marker, lane 1; Positive control (*Orientia*, *Leptospira*, and *Rickettsia* DNA mixture), lane 2; *O*. *tsutsugamushi* (166 bp), lane 3; *L*. *interrogans* (243 bp), lane 4; *R*. *typhi* (434 bp), lane 5; *E*. *faecalis*, lane 6; *S*. *aureus*, lane 7; *Salmonella* spp., lane 8; *K*. *pneumoniae*, lane 9; Dengue virus I, lane 10; Dengue virus II, lane 11; Dengue virus III, lane 12; Dengue virus IV, lane 13; *P*. *falciparum*, lane 14; *P*. *vivax*, lane 15; *E*. *coli*, and lane 16; negative control. (B) Multiplex PCR was tested in masking condition. Lane M; 100bp marker, lane 1; Positive control (*Orientia*, *Leptospira*, and *Rickettsia* DNA mixture), lane 2; DNA mixture of *O*. *tsutsugamushi*, *S*. *aureus*, and dengue virus, lane 3; DNA mixture of *L*. *interrogans*, *S*. *aureus*, and dengue virus, lane 4; DNA mixture of *R*. *typhi*, *S*. *aureus*, and dengue virus, lane 5; negative control.

To determine the specificity of multiplex PCR, we tested three primer pairs with other pathogens, including *E*. *faecalis*, *S*. *aureus*, *Salmonella* spp., *K*. *pneumoniae*, *E*. *coli*, Dengue viruses, *P*. *falciparum*, and *P*. *vivax*. No amplification was obtained from unrelated pathogens (Fig 2A). In addition, individual primer pair was able to amplify each target gene in the masking condition (Fig 2B).

### Sensitivity of multiplex PCR assay

**Fig 3:**
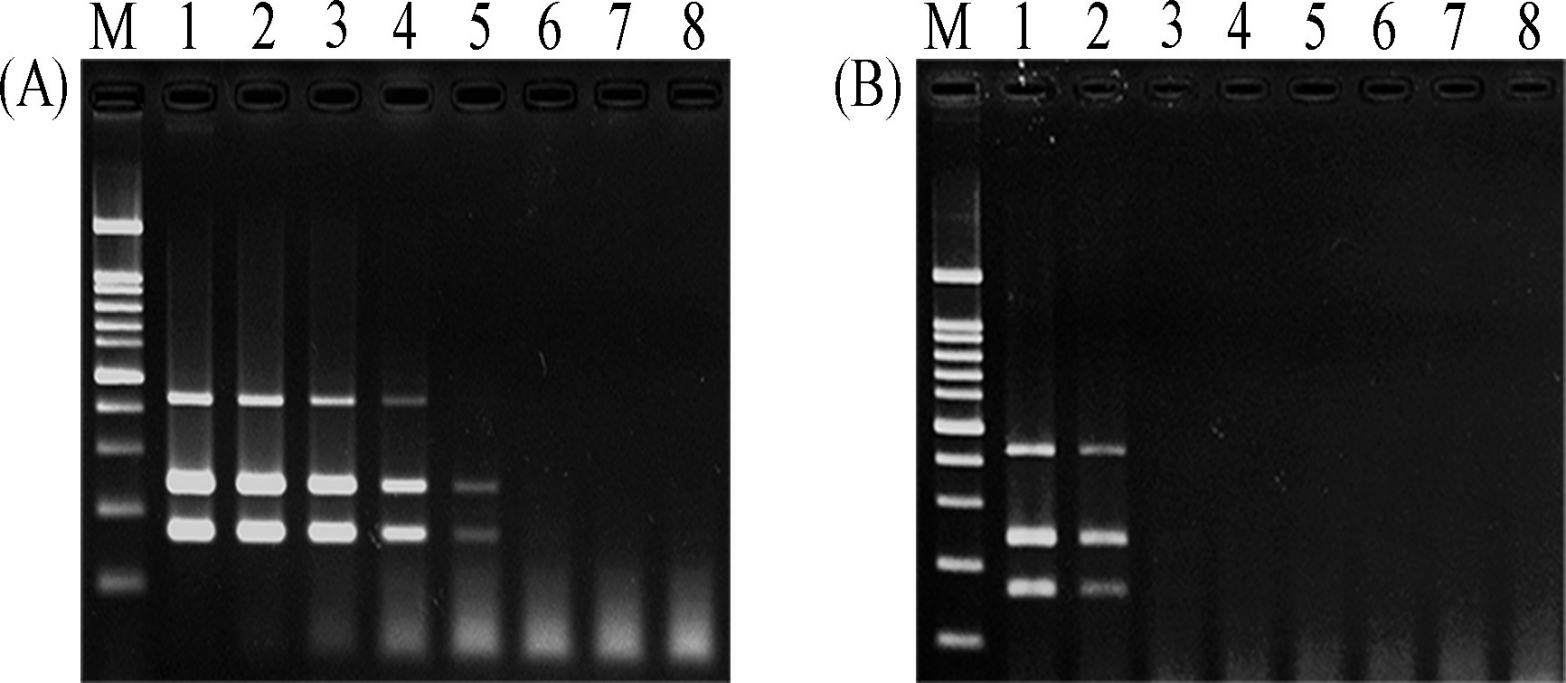
Sensitivity of multiplex PCR assay. (A) Limit of detection of multiplex PCR with a serial dilution of *Orientia*, *Leptospira*, and *Rickettia* DNA mixture, and (B) limit of detection with three bacterial DNA-spiked blood sample in different concentration. In both figures: lane M; 100bp marker, lane 1; 5 ng/μl, lane 2; 0.5 ng/μl, lane 3; 50 pg/μl, lane 4; 5 pg/μl, lane 5; 0.5 pg/μl, lane 6; 50 fg/μl, lane 7; 5 fg/μl, and lane 8; negative control.

In this experiment, we evaluated limit of detection using tenfold serial dilution, ranging from 5x10^0^ to 5x10^-6^ng/μl per reaction, of mixed DNA and mixed DNA-spiked blood samples. The results have shown that the lowest concentrations of mixed DNA were 0.5pg/μl or 230 copies for *O*. *tsutsugamushi*, 0.5pg/μl or 106 copies for *L*. *interrogans*, and 5pg/μl or 4,160 copies for *R*. *typhi* (Fig 3A). In contrast, the detection limit of mixed DNA-spiked blood samples was 0.5ng/μl for all targets (Fig 3B).

### Performance of multiplex PCR assay

**Fig 4:**
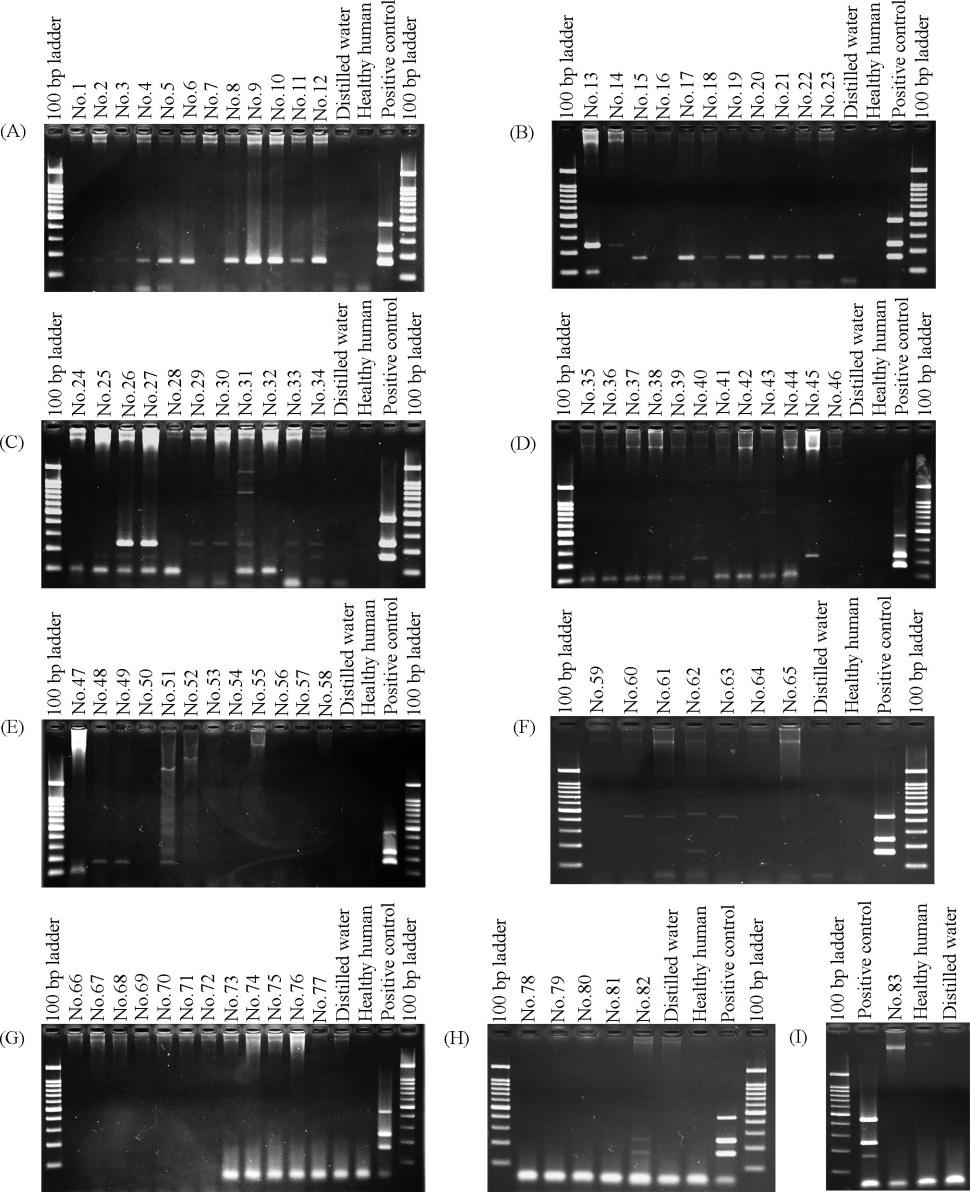
Performance of the multiplex PCR. (A-H) showed the multiplex PCR results when tested with blood specimens from Thai patients presented with acute undifferentiated febrile illness.

To validate efficiency of multiplex PCR assay, we performed multiplex PCR with 83 EDTA blood samples obtained from Thai patients presented with acute undifferentiated febrile illness. Multiplex PCR results were positive in 39 samples (47%), consisting of 22 samples of scrub typhus (26.5%), 11 samples of leptospirosis (13.25%), 5 samples of murine typhus (6.02%), and 1 sample of co-infection between scrub typhus and leptospirosis (1.2%). For comparison, standard serological methods results were positive in 20 samples (24.09%). The results are shown in Table 3 and Fig 4.

**Table 3:** Comparison of multiplex PCR results and standard serological test results.

Methods/Results			Serological test (either IFA or MAT test)		Total
			Positive	Negative	
**Multiplex PCR**	**Positive**	**Scrub typhus**	13	9	39
		**leptospirosis**	6	5	
		**Murine typhus**	1	4	
		**Co-infection**	0	1	
	**Negative**		0	44	44
**Total**			20	63	83

Twenty samples were detected by both serological methods and multiplex PCR. Nineteen samples were detected only by multiplex PCR but tested negative by standard serological methods. In addition, 1 in 39 samples exhibited co-infection between scrub typhus and leptospirosis. The diagnostic sensitivity and specificity of multiplex PCR were 100% and 70%, respectively, when serological methods were used as gold standard. Positive predictive value and negative predictive value of this assay were 51% and 100%, respectively.

## Discussion

To our knowledge, this work is the first study to combine 3 primer pairs targeting 56-kDa TSA gene of *O*. *tsutsugamushi*, 17-kDa antigen gene of *R*. *Typhi*, and lipL32 gene of *L*. *interrogans*. Our results showed that this multiplex PCR assay was able to specifically detect each bacterial DNA target. The primer sets did not cross-react with other bacteria. Moreover, we validated this assay using EDTA blood samples from patients with acute undifferentiated febrile illness. Sensitivity and specificity of the multiplex PCR assay developed in this study were 100% and 70%, respectively.

In the present, standard diagnostic tests for common causes of acute differentiated fever in Thailand are based on antibody detection, such as MAT for leptospirosis and IFA for both scrub typhus and murine typhus. However, these methods require paired sera, extensive labor, and specialized facilities only available at reference laboratories [18, 21]. The delayed diagnosis and treatment may result in severity, complication, and mortality. Therefore, a rapid diagnostic test is necessary for early pathogen detection. Multiplex PCR can be completed within 5 hours, reducing turnaround time for pathogen identification [22, 23]. Previous studies reported conventional PCR, nested PCR, real-time PCR, and loop-mediated isothermal amplification (LAMP) for diagnosis of scrub typhus, murine typhus, and leptospirosis [2, 24]. However, multiplex PCR for simultaneous detection of these three neglected tropical diseases has never been reported. Multiplex PCR is faster and cheaper than conventional PCR for individual disease and serological methods [25]. In addition, this assay is easier and more convenient than nested PCR as well as LAMP. Multiplex PCR also reduces the risk of contamination between amplification [22]. Thus, multiplex PCR can be a practical and reliable diagnostic tool for multiple pathogens detection.

Here, we developed multiplex PCR assay that could be used for diagnosis of three neglected diseases in a single tube. Our results have shown that the extension temperature of 68°C gave optimal result compared to 72°C. These bacterial DNA are highly AT-rich (65–70%), so the reduction in extension temperature can greatly increase PCR amplification for AT-rich templates [26–29]. On the other hand, extension temperature at 72°C prevents DNA synthesis at highly AT-rich region [30]. Because having multiple primer pairs in a single tube negatively affect amplification efficiency, further optimization is crucial. Optimal Mg^2+^ concentration is necessary for *Taq* DNA polymerase and specificity of primer-template binding. Inadequate Mg^2+^ concentration decreases PCR product yield and specificity due to incorrect primer-template binding [31]. The Mg^2+^ concentration for our assay was optimal at a final concentration of 2.5 mM. Using a gradient thermal cycler, annealing temperature was optimized for sensitivity and specificity of primer-template binding [32]. Although AT-rich templates require lower annealing temperature [28], 61°C proved to be optimal for this multiplex amplification.

The limit of detection of our multiplex PCR were 0.5 pg/μl or 230 copies for *O*. *tsutsugamushi*, 0.5 pg/μl or 106 copies for *L*. *interrogans*, and 5 pg/μl or 4,160 copies for *R*. *typhi*. Detection limits of this multiplex PCR were comparable to that of previous studies that detected *O*. *Tsutsugamushi*, *R*. *Typhi*, and *L*. *Interrogans* using singleplex PCR [5, 33, 34]. The limit of detection of multiplex PCR depends on the followings: 1) combination of multiple primers in a single tube that might affect effective amplification; 2) product size, because amplification is more effective in case of smaller product [15, 35]. The higher limit of detection in mixed bacterial DNA-spiked blood samples, 0.5ng/μl, might be a result of PCR inhibitors, such as hemoglobin and other components in blood [36, 37].

*Orientia* and *Rickettsia* are obligate intracellular bacteria that can be found in buffy coat of blood specimens [2]. However, buffy coat is more difficult to collect than blood samples. Fortunately, leptospires could be found in bloodstream during the acute phase [18]. Previous studies had also reported detection of *Orientia*, *Rickettsia*, and *Leptospira* DNA in blood samples [2, 38]. In this study, we used EDTA blood samples for multiplex PCR validation. Thirty-nine samples (47%) were found to be positive by multiplex PCR, whereas 20 samples (24.09%) were positive by standard serological tests. Nineteen of the 39 samples, which tested positive in multiplex PCR analysis, exhibited negative serological results. These results may be explained by the period of blood collection and the phase of diseases. In the early phase of infection, bacteremia might not induce sufficient antibody level for serological detection [39–41]. Our study suggests that these 19 blood samples were bacteremic and, inferably, collected during the early phase of infection. Consequently, the multiplex PCR was able to detect bacterial DNA in the blood specimens, even though serological tests showed negative results. One sample exhibited dual positive of *O*. *tsutsugamushi* and *L*. *interrogans* in multiplex PCR assay, while tested negative in serological analyses. Previous studies had reported co-infection between *O*. *tsutsugamushi* and pathogenic *Leptospira* spp. in Thai and Taiwanese patients with acute undifferentiated fever [42, 43]. This study suggests that multiplex PCR can be applied in clinical diagnostic test for identifying pathogenic agents that cause scrub typhus, leptospirosis, murine typhus, and co-infections.

This multiplex PCR is a useful tool for early diagnosis of scrub typhus, murine typhus, and leptospirosis. Moreover, the combination of multiplex PCR and serological assays helps increase sensitivity for diagnosis and confirmation. However, sensitivity and specificity determined in this study are only preliminary measurements due to limited sample size. Further study should be performed using a large number of clinical samples.

In conclusion, we developed a novel multiplex PCR assay for identifying causative agents of scrub typhus, murine typhus, and leptospirosis in blood samples. This method is a rapid, sensitive, and specific diagnostic test. The multiplex PCR assay will become useful for the development of better health care and treatment of patients presented with acute undifferentiated febrile illness, particularly in endemic areas of these diseases.
